# Comparative Proteomic Analysis of Coregulation of CIPK14 and WHIRLY1/3 Mediated Pale Yellowing of Leaves in *Arabidopsis*

**DOI:** 10.3390/ijms19082231

**Published:** 2018-07-31

**Authors:** Zhe Guan, Wanzhen Wang, Xingle Yu, Wenfang Lin, Ying Miao

**Affiliations:** Center for Molecular Cell and Systems Biology, Fujian Provincial Key Laboratory of Haixia Applied Plant Systems Biology, College of Life Sciences, Fujian Agriculture and Forestry University, Fuzhou 350002, China; 1150539002@fafu.edu.cn (Z.G.); 1150539007@fafu.edu.cn (W.W.); 17720802384@163.com (X.Y.); linwf@fafu.edu.cn (W.L.)

**Keywords:** comparative proteomic analysis, CIPK14, WHIRLY1/WHIRLY3, protein metabolism

## Abstract

Pale yellowing of leaf variegation is observed in the mutant *Arabidopsis* lines Calcineurin B-Like-Interacting Protein Kinase14 (CIPK14) overexpression (*oeCIPK14*) and double-knockout *WHIRLY1/WHIRLY3* (*why1/3*). Further, the relative distribution of WHIRLY1 (WHY1) protein between plastids and the nucleus is affected by the phosphorylation of WHY1 by CIPK14. To elucidate the coregulation of CIPK14 and WHIRLY1/WHIRLY3-mediated pale yellowing of leaves, a differential proteomic analysis was conducted between the *oeCIPK14* variegated (*oeCIPK14-var*) line, *why1/3* variegated (*why1/3-var*) line, and wild type (WT). More than 800 protein spots were resolved on each gel, and 67 differentially abundant proteins (DAPs) were identified by matrix-assisted laser desorption ionization-time of flight/time of flight mass spectrometry (MALDI-TOF/TOF-MS). Of these 67 proteins, 34 DAPs were in the *oeCIPK14-var* line and 33 DAPs were in the *why1/3-var* line compared to the WT. Five overlapping proteins were differentially expressed in both the *oeCIPK14-var* and *why1/3-var* lines: ATP-dependent Clp protease proteolytic subunit-related protein 3 (ClpR3), Ribulose bisphosphate carboxylase large chain (RBCL), Beta-amylase 3 (BAM3), Ribosome-recycling factor (RRF), and Ribulose bisphosphate carboxylase small chain (RBCS). Bioinformatics analysis showed that most of the DAPs are involved in photosynthesis, defense and antioxidation pathways, protein metabolism, amino acid metabolism, energy metabolism, malate biosynthesis, lipid metabolism, and transcription. Thus, in the *why1/3-var* and *oeCIPK14-var* lines, there was a decrease in the photosystem parameters, including the content of chlorophyll, the photochemical efficiency of photosystem (PS II) (F_v_/F_m_), and electron transport rates (ETRs), but there was an increase in non-photochemical quenching (NPQ). Both mutants showed high sensitivity to intense light. Based on the annotation of the DAPs from both *why1/3-var* and *oeCIPK14-var* lines, we conclude that the CIPK14 phosphorylation-mediated WHY1 deficiency in plastids is related to the impairment of protein metabolism, leading to chloroplast dysfunction.

## 1. Introduction

Leaf senescence is a complex process that is highly regulated by genetic material, and is induced by internal (such as age and hormones) and external (including multiple biotic and abiotic stresses) factors [1]. Leaf senescence results from the degradation of chlorophyll and various biomacromolecules in plant cells [1] for the remobilization of nutrients to seeds and fruits during the reproductive growth stage [2]. It has been reported that more than 20 transcription factor families are associated with senescence regulation, such as the NAC, WRKY, MYB, C2H2-type zinc finger, and AP2/EREBP protein families. Many members of the NAC and WRKY families have been reported to play a central role in the regulatory network that controls leaf senescence [3,4,5,6,7]. Among the WRKY family, *WRKY53* has been shown to act as a key regulator at an early stage of leaf senescence in *Arabidopsis* [8], and WHIRLY1 has been reported to repress the expression of *WRKY53* by binding to the promoter of *WRKY53*, thus delaying leaf senescence [9].

WHIRLY (WHY) family proteins are located in both the nucleus and organelles, and they perform numerous cellular functions at both sites [10,11]. There are two WHY members (WHY1 and WHY2) in monocotyledonous plants, and three members (WHY1, WHY2, and WHY3) in dicotyledonous plants [12]. A WHY–GFP fusion protein fluorescence assay showed that, in *Arabidopsis*, WHY1 and WHY3 localized to chloroplasts, and WHY2 localized to mitochondria [10]. An intriguing feature of WHY1’s dual location in plastids and the nucleus of the same cell was demonstrated by an immunogold-labeling assay in barley (*Hordeum vulgare*) [11]. Plastidial WHY1 translocated from chloroplasts to the nucleus in transplastomic tobacco plants, which synthesized an HA-tagged version of WHY1 in plastids. In the nucleus, the WHY1 protein changes the expression of its target genes [13]. Its dual location and ability to regulate nuclear gene transcription make WHY1 an ideal candidate for studying plastid-to-nucleus retrograde signaling.

WHY proteins were first discovered as nuclear transcriptional activators binding at the elicitor response element in the promoter regions of pathogenesis-related genes in potato (*Solanum tuberosum*) and *Arabidopsis* [14,15]. It was later found that these proteins bind to various DNA sequences, including: telomeres [16], a distal element upstream of a *kinesin* gene [17], the promoter region of the early senescence marker gene *WRKY53* (in a development-dependent manner) in *Arabidopsis* [9], and the promoter region of the senescence-associated gene *HvS40*, which was induced during natural and stress-related senescence in barley [18]. It has further been proposed that WHY1 binds to both single stranded DNA (ssDNA) and RNA in maize (*Zea mays*) chloroplasts, where it plays a role in intron splicing [19], and WHY1 is associated with intron-containing RNA in barley chloroplasts [20].

Additionally, in *Arabidopsis*, plastid-located WHY1 and WHY3 were identified as two novel plastid transcriptionally active chromosome proteins (pTACs) by mass spectrometry (MS) in the transcriptionally active chromosomes of nucleoids [21]. Moreover, both WHY3 and WHY1 were found in a protein complex bound to the promoter of *kinesin* in *Arabidopsis* by a pull-down-MS assay [17]. While WHY3 was discovered as a redox-affected protein in the thiol–disulfide redox proteome of the chloroplast [22], WHY1 was proposed to be involved in the perception of redox changes in the photosynthetic apparatus. Thus, the relocation of WHY1 from the chloroplasts to the nucleus may be initiated by the redox state of the photosynthetic electron transport chain [23]. A recent study indicated that WHY1 interacts with light-harvesting protein complex I (LHCA1) and affects the expression of genes encoding photosystem I (PS I) and light-harvesting complexes (LHCIs) [24]. Although most double-knockout *WHY1* and *WHY3* (*why1*/*3*) plants have no apparent phenotype, about 5% of the plants show a variegated phenotype, which is associated with the instability of the plastid genome [25]. Furthermore, triple-mutant *why1why3polIb-1* (defective WHY1, WHY3, and chloroplast DNA polymerase 1B (POLIB)) exhibits a significant variegated phenotype and higher plastid genome instability [26]. The *why1why3polIb-1* mutant shows lower photosynthetic efficiency and produces more reactive oxygen species (ROS) in the chloroplast, and the elevated ROS level is correlated with an elevated expression of oxidation-related nuclear genes [26]. In barley, *WHY1* RNAi knockdown mutants were shown to have more chlorophyll and less sucrose than the wild type [27]. A large number of gene-encoding proteins involved in photosynthesis and protein synthesis are upregulated in *Hvwhy1* mutants [27]. These results suggest that plastid-located WHY proteins participate in plastid-to-nucleus retrograde signaling to maintain plastid function in response to environmental fluctuations. Their dual location and dual function suggest that WHY proteins have special traits for communicating between the two compartments in one cell. 

The latest study illuminated that Calcineurin B-like-Interacting Protein Kinase14 (CIPK14) interacts with and phosphorylates WHY1, and phosphorylated WHY1 is imported to the nucleus with an enhanced binding affinity for the promoter of *WRKY53* [28]. *CIPK14* overexpression (*oeCIPK14*) transgenic plants show an increased nuclear isoform and decreased plastid isoform of WHY1. Fascinatingly, about 5% of *CIPK14* overexpression transgenic plants show a variegated pale-green phenotype, which is similar to the *why1/3* and *why1why3polIb-1* mutants [28]. Even more intriguing, the variegated phenotype can be partially recovered by the overexpression of plastid-located WHY1 [28]. 

This study focuses on the comparable analysis of the phenotypic and proteomic alterations between the *why1/3* variegated (*why1/3-var*) lines and oe*CIPK14* variegated (*oeCIPK14-var*) lines to evaluate the relationship between CIPK14 and WHY1/WHY3, and to determine their role in producing pale yellow leaves. Total protein in rosette leaves from wild type (WT), *why1/3-var*, and *oeCIPK14-var* was separated by two-dimensional gel electrophoresis analysis (2-DE), and the differentially expressed proteins were identified by matrix-assisted laser desorption ionization-time of flight/time of flight mass spectrometry (MALDI-TOF/TOF-MS). The selected proteins were identified at the transcriptional level by quantitative real-time PCR (qRT-PCR) and at the protein level by Western blot. The chlorophyll content and chlorophyll fluorescence kinetic curve were used to determine the photosynthetic performance related to the phenotypes of different mutants.

## 2. Results

### 2.1. Proteomic Analysis of why1/3-var and oeCIPK14-var Mutants

**Figure 1:**
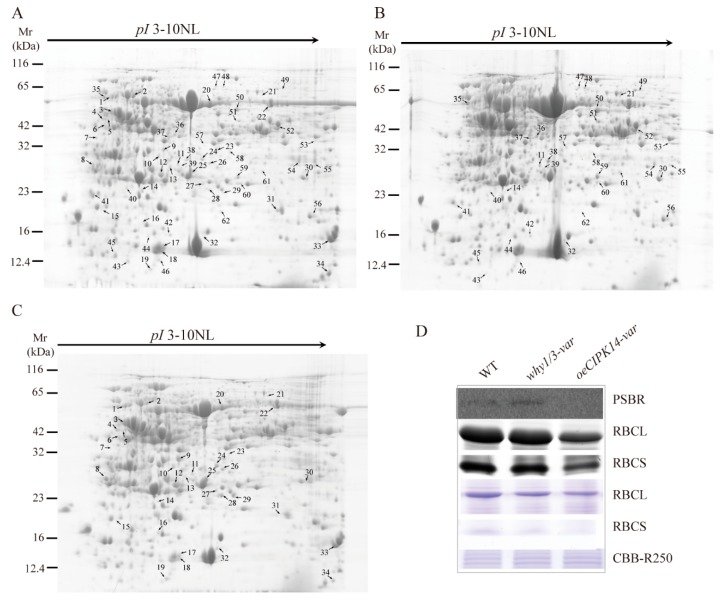
2-DE and immunoblot analysis of total proteins extracted from rosette leaves of WT and two variegated mutants. (**A**–**C**) Representative 2-DE gel images of WT (**A**); *why1/3-var* (**B**); and *oeCIPK14-var* (**C**). An equal amount (1.5 mg) of total proteins was loaded on each IPG strips (3–10 NL). The spot numbers indicated proteins that showed significant changes between WT and two variegated mutants. (**D**) The changing of protein abundance selected from 2-DE were confirmed by western blot and CBB R250 staining. The immunoblot analysis is performed using antibodies against RBCL, RBCS, and PSBR. CBB R250 staining shows RBCL and RBCS protein amount and the same amount of loading proteins.

Based on our previous reports, the dual localization and distribution of the WHY1 protein in plastids and the nucleus are affected by the WHY1 protein’s phosphorylation status, which is mediated by CIPK14. Of the *CIPK14* overexpression transgenic plants, about 5% of the *oeCIPK14-var* line showed the variegated pale-green phenotype, which is similar to the *why1/3-var* phenotype [28]. To evaluate the relationship between CIPK14 and WHY1/WHY3 at the protein level and their association with the production of pale yellow leaves, proteomic analysis was conducted. The total protein in rosette leaves of 4-week-old plants was separated by 2-DE; more than 800 protein spots were detected reproducibly on each gel for the WT, *why1/3-var*, and *oeCIPK14-var* lines. A total of 67 differentially expressed protein spots showed significant changes (fold > 2, *p* < 0.05) in the *why1/3-var* and *oeCIPK14-var* lines (Figure 1A–C). The differentially expressed protein spots were analyzed by MALDI-TOF/TOF-MS, and a total of 66 protein spots were identified successfully (identification rate: 99%); only one protein (spot 42) remains unknown. The identified proteins are listed in Table 1. Among them, 33 differentially expressed protein spots (spots 11, 14, 21, 30, 32, 35–62) were detected in *why1/3-var* (Figure 1B), and 34 differentially expressed protein spots (spots 1–34) were detected in *oeCIPK14-var* (Figure 1C). Interestingly, only five overlapping proteins (spots 11, 14, 21, 30, 32) were discovered in both *why1/3-var* and *oeCIPK14-var*. These five proteins are proposed to be intimately associated with the CIPK14-mediated functions of WHY proteins.

In order to evaluate the quality of the 2-DE differentially displayed proteins, two light-dependent reaction complex-related proteins were selected for immunodetection, using consumable antibodies against ATP synthase subunit alpha (atp A) and the photosystem II (PS II) complex protein PSBR (Figure 1D). While the antibody against atp A did not produce any reaction, PSBR was barely detectable in the *oeCIPK14-var* line, which is in agreement with the changes in protein abundance observed by 2-DE (Figure 1D). We further directly detected the levels of Ribulose bisphosphate carboxylase large chain (RBCL) and Ribulose bisphosphate carboxylase small chain (RBCS) by Coomassie bright blue R250 staining (Figure 1D), which clearly showed that both RBCL and RBCS decreased in the *why1/3-var* and *oeCIPK14-var* lines, consistent with the changes in protein abundance observed by 2-DE. Furthermore, two antibodies against RBCL and RBCS were used to perform Western blotting. Consistent with our 2-DE findings, these results showed that the proteins RBCL and RBCS were downregulated in the *oeCIPK14-var* line and slightly decreased in the *why1/3-var* plants, as compared to wild-type plants (Figure 1D). These findings confirm that the MS data are reliable.

### 2.2. Functional Classification of Differentially Expressed Proteins

**Figure 2:**
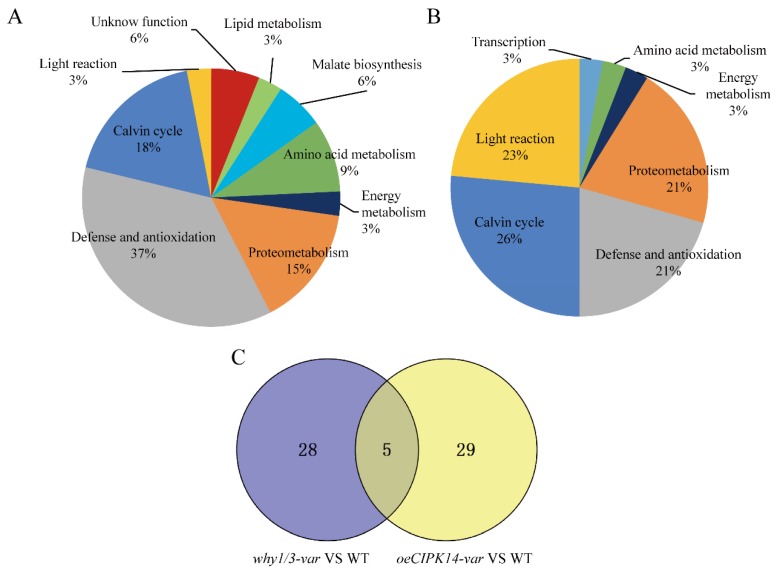
Functional classifications of the differentially expressed proteins identified in *why1/3-var* and *oeCIPK14-var* compared with WT. (**A**) Functional classifications of the differentially expressed proteins between *why1/3-var* and WT; (**B**) Functional classifications of the differentially expressed proteins between *oeCIPK14-var* and WT; (**C**) The Venn diagram analysis between *why1/3-var* and *oeCIPK14-var* compared with WT. The Venn diagram is completed by the online tool (available online: http://bioinfogp.cnb.csic.es/tools/venny/index.html).

Based on the Uniprot database (http://www.uniprot.org/) and descriptions from the literature, the functions of 65 proteins were annotated. These proteins are categorized into eight groups based on their biochemical functions, as shown in Table 1. The majority of the proteins are photosynthesis-associated proteins, followed by proteins related to defense and antioxidation, protein and amino acid metabolism, energy metabolism, malate biosynthesis, lipid metabolism, and transcription (Table 1 and Figure 2). Comparative analysis of differentially expressed proteins between *why1/3-var*/WT and *oeCIPK14-var*/WT are shown in Figure 2A,B, respectively. The five overlapping proteins between *why1/3-var*/WT and *oeCIPK14-var*/WT are ATP-dependent Clp protease proteolytic subunit-related protein 3 (ClpR3), Ribulose bisphosphate carboxylase large chain (RBCL), Beta-amylase 3 (BAM3), Ribosome-recycling factor (RRF), and Ribulose bisphosphate carboxylase small chain (RBCS) (Figure 2C).

### 2.3. Hierarchical Clustering Analysis of Differentially Expressed Proteins

**Figure 3:**
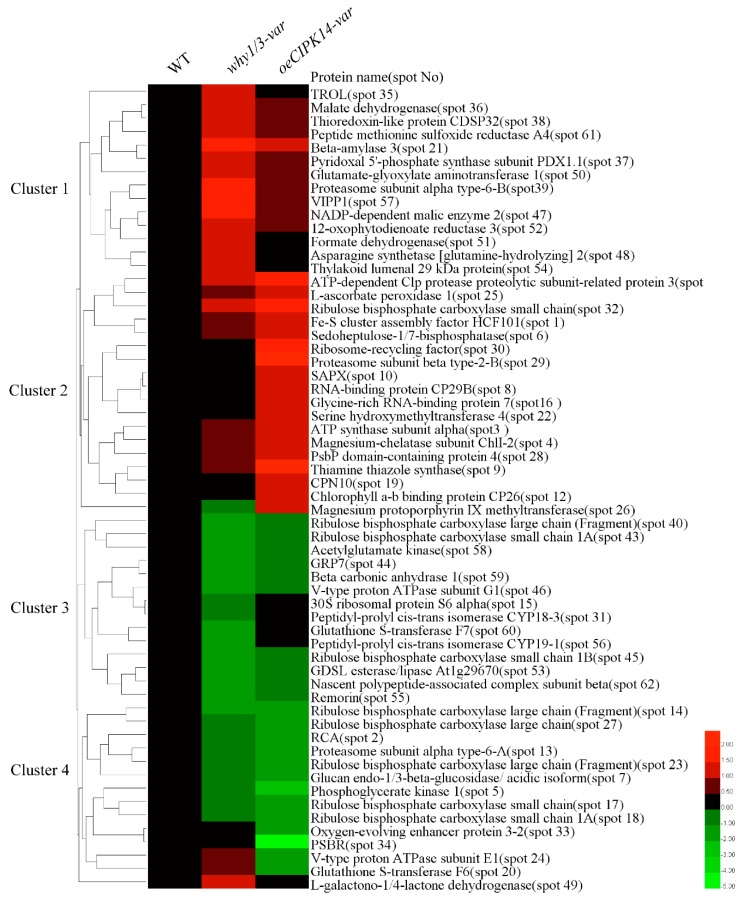
Hierarchical cluster analysis of the differentially expressed proteins of *why1/3-var* and *oeCIPK14-var* compared with WT. The three columns represent protein expression changings in the (**A**) WT, (**B**) *why1/3-var*, and (**C**) *oeCIPK14-var*, respectively. The rows represent individual proteins identified in the *why1/3-var* and *oeCIPK14-var* lines; the up-regulated or down-regulated proteins are indicated in red or green. The heat map used log2 of fold changes of protein abundance between WT and *why1/3-var* and *oeCIPK14-var* mutants.

To acquire information on the identified proteins, hierarchical clustering analysis was performed on the proteins appearing on the same branch with similar expression patterns (Figure 3). There are four clusters of these proteins. The majority of the proteins in the first and second clusters were upregulated in the *why1/3-var* and *oeCIPK14-var* lines, respectively, and mainly contain defense and antioxidation-related proteins (spots 9, 10, 25, 37, 38, 52, 54, 57, 61), amino acid and protein metabolism-related proteins (spots 11, 19, 22, 29, 30, 39, 48, 50), photosynthesis-associated proteins (spots 1, 3, 4, 6, 12, 26, 28, 32, 35), and energy metabolism-related proteins (spots 21, 36, 47, 51). Most of the proteins in the third and fourth clusters were downregulated in the *why1/3-var* and *oeCIPK14-var* lines, respectively. Among them, some of the key enzymes of the Calvin cycle (spots 2, 5, 6, 14, 17, 18, 23, 27, 40, 43, 45) belong to the two branches, suggesting that the fixation rates of CO_2_ decrease in the *why1/3-var* and *oeCIPK14-var* variegated lines. We also found that other proteins downregulated in the *why1/3-var* and *oeCIPK14-var* lines are involved in light reaction, defense and antioxidation mechanisms, amino acid metabolism, protein metabolism, and so on.

### 2.4. Transcriptional Level Analysis of the Encoding Genes of Differentially Expressed Proteins

**Figure 4:**
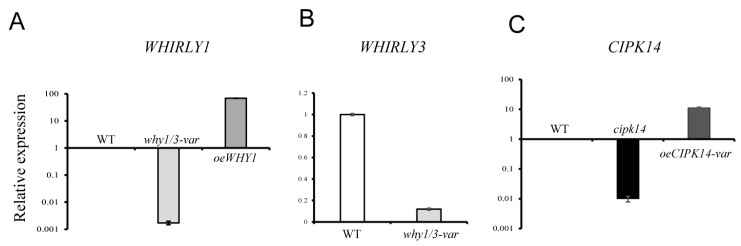
The transcript levels of *WHIRLY1*, *WHIRLY3* and *CIPK14* in the used lines. (**A**) The level of expression of *WHIRLY1* in the WT, *why1/3-var* and *oeWHY1*; (**B**) The level of expression of *WHIRLY3* in the WT and *why1/3-var*; (**C**) The level of expression of *CIPK14* in the WT, *cipk14* and *oeCIPK14-var*.

To investigate the correlation between protein and transcript levels, the transcript levels of the encoding genes of differentially expressed proteins were analyzed by quantitative real-time PCR (qRT-PCR). Twenty-six genes were identified in the WT, *why1/3-var*, and overexpressing WHY1 (*oeWHY1)* lines; and 27 genes were identified in the WT, *oeCIPK14-var*, and knockout *CIPK14 (cipk14)* lines, respectively. The transcript levels of WHY1, WHY3, and CIPK14 were first detected in the WHY and CIPK14 mutants (Figure 4). As shown in Figure 4, the expression level of *WHIRLY1* was barely detectable in the *why1/3-var* line, but it was significantly accumulated in *oeWHY1* (Figure 4A). The expression level of *WHIRLY3* was lower in *why1/3-var* compared with WT (Figure 4B). The expression level of *CIPK14* was barely detectable in *cipk14*, while it showed significant upregulation in *oeCIPK14-var* (Figure 4C).

**Figure 5:**
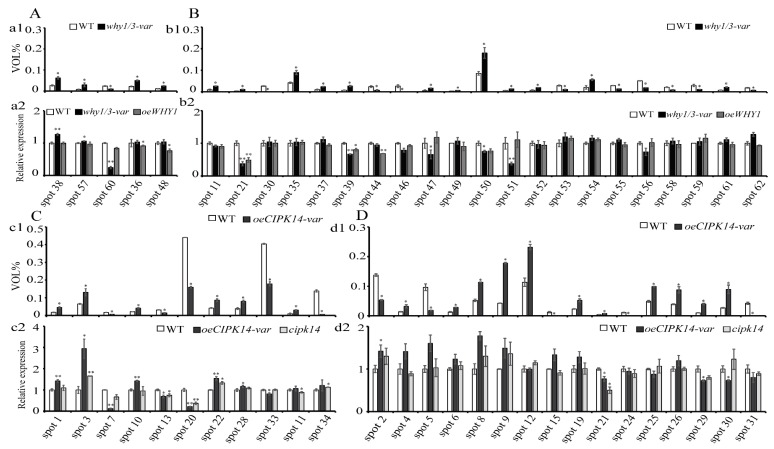
Comparison of changes in the protein and mRNA levels for selected protein spots. The relative protein abundance is represented by percent volume. (**A**) The mRNA levels change in parallel with protein levels in the *why1/3-var* lines; (**a1**) The relative protein abundance of protein spots in the WT and *why1/3-var*; (**a2**) The relative expression levels of the corresponding genes in (**a1**) are analyzed in the WT, *why1/3-var*, and *oeWHY1*; (**B**) The mRNA levels change independently in the *why1/3-var* lines; (**b1**) The relative protein abundance of protein spots in the WT and *why1/3-var*; (**b2**) The relative expression levels of the corresponding genes (**b1**) are analyzed in the WT, *why1/3-var*, and *oeWHY1*; (**C**) The mRNA levels change in parallel with protein levels in the *oeCIPK14-var* lines; (**c1**) The relative protein abundance of protein spots in the WT and *oeCIPK14-var*; (**c2**) The relative expression level of the corresponding genes in (**c1**) are analyzed in the WT, *oeCIPK14-var*, and *cipk14*; (**D**) The mRNA levels change independently in the *oeCIPK14-var* lines; (**d1**) The relative protein abundance of protein spots in the WT and *oeCIPK14-var*; (**d2**) The relative expression of the corresponding genes in (**d1**) are analyzed in the WT, *oeCIPK14-var*, and *cipk14*. The relative expression level of the gene is normalized to *GAPC2*, with the WT as 1. The error bars indicated standard error of three biological replications and three technique replicates. Asterisk indicate significant differences (* *p* < 0.05 and ** *p* < 0.01) based on Student’s *t*-test analyzed by Graphpad prism6 software.

We further analyzed the transcript levels of the encoding genes of differentially expressed proteins. Relative protein abundance of WT and *why1/3-var* is represented by percent volume on the top panels (a1 and b1) of Figure 5A,B, and the relative expression levels of the encoding genes of differentially expressed proteins in *why1/3-var* and *oeWHY1* compared to WT are shown in Figure 5A(a2),B(b2), respectively. The encoding genes of differentially expressed proteins in *oeCIPK14-var* were analyzed in the WT, *cipk14*, and *oeCIPK14-var* lines. Relative protein abundance in WT and *oeCIPK14-var* is represented by percent volume in the top panels (c1 and d1) of Figure 5C,D, and the relative expression levels of the encoding genes of differentially expressed proteins in *oeCIPK14-var* and *cipk14* compared to WT are shown in Figure 5C(c2),D(d2), respectively. The quantitative RT-PCR results of all candidate genes showed that the transcript levels of 11 of the 26 genes were altered in *why1/3-var* or *oeWHY1*, and the transcriptional patterns of 5 of the 26 genes are consistent with the protein expression patterns (Figure 5A(a2)). The transcripts of the remaining 21 genes are inconsistent with protein levels in *why1/3-var* (Figure 5B(b2)). However, most of the changes in gene expression in *why1/3-var* were recovered in *oeWHY1*, except for spot 21, spot 39, and spot 44. This suggests that WHY1 is not sufficient to fully rescue the alteration of transcript levels of genes in *why1/3-var*. The transcript levels of 13 of the 27 expressed genes were changed in *oeCIPK14-var* or *cipk14*, and the mRNA levels of 9 of the 27 genes changed in parallel with protein levels (Figure 5C(c2)). The mRNA levels of the remaining 18 genes were inconsistent with *oeCIPK14-var* protein levels (Figure 4D(d2)). Unexpectedly, only a few gene expression levels were changed in the *cipk14* line, and none of the changes in gene expression were rescued. This discrepancy between mRNA and protein levels may be caused by the different half-lives of protein and mRNA, or post-transcriptional or post-translational processing and modification.

The relative expression level of the gene is normalized to *GAPC2*, with the WT as 1. The error bars indicated standard error of three biological replications and three technique replicates. Asterisks indicate significant differences (* *p* < 0.05 and ** *p* < 0.01) based on Student’s *t*-test analyzed by Graphpad prism6 software.

### 2.5. Effects of why1/3-var and oeCIPK14-var on Photosynthetic Performance

**Figure 6:**
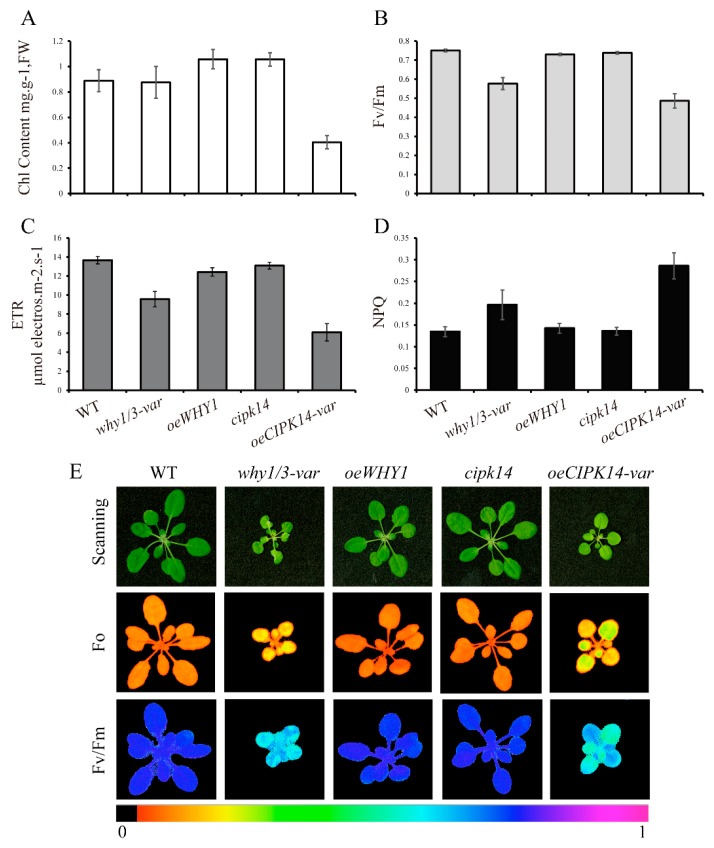
The photosynthetic performance analysis of WHY and CIPK14 mutants. (**A**) Total chlorophyll content; (**B**) The maximum photochemical efficiency of photosystem II (F_v_/F_m_); (**C**) The nonphotochemical quenching of photosystem II fluorescence (NPQ); (**D**) The electron transport rate (ETR). The error bars indicate the standard error of nine independent measurements; (**E**) The fluorescence images of the whole plants of WHY and CIPK14 mutants. The fluorescence images are taken by Image-PAM using the plants after being dark-adapted 30 min.

According to the above protein annotations, the majority of the differentially expressed proteins are photosynthesis-associated proteins. In order to address the relationship between protein function and the pale-green leaf phenotype in the *why1/3-var* and *oeCIPK14-var* lines, the chlorophyll contents and photosynthetic fluorescence parameters of 4-week-old plants of *WHY* and *CIPK14* mutants were measured to determine the photosynthetic performance. Consistent with the pale-green phenotype, the chlorophyll content was lower in the *oeCIPK14-var* plants than in the wild type, whereas it remained unaltered in the *why1/3-var* line (Figure 6A). Interestingly, the chlorophyll content slightly increased in the *oeWHY1* and *cipk14* plants (Figure 6A). The maximum photochemical efficiency of photosystem II (F_v_/F_m_) significantly decreased in the *why1/3-var* and *oeCIPK14-var* plants (Figure 6B). The electron transport rates (ETRs) in *why1/3-var* and *oeCIPK14-var* were only about 70% and 45% of the WT, respectively (Figure 6C). The nonphotochemical quenching (NPQ) of photosystem II fluorescence displayed an increase in *why1/3-var* and *oeCIPK14-var* (Figure 6D), indicating that the two variegated mutants have a lower photosynthetic capacity, which is consistent with plants under high light conditions.

Fluorescence images of whole plants with different genotypes were taken using an Image-PAM (Pulse-Amplitude Modulation) measuring system, as shown in Figure 6E. The *why1/3-var* and *oeCIPK14-var* plants displayed a smaller size and variegated phenotype, as described previously [25,28]. While the *oeWHY1* and *cipk14* plants were almost indistinguishable from the wild type, the dark fluorescence yield (F0) of the two variegated lines was higher, but the F_v_/F_m_ decreased markedly in the *why1/3-var* and *oeCIPK14-var* lines.

Taken together, in the *why1/3-var* and *oeCIPK14-var* plants, higher F0, lower F_v_/F_m_, lower ETR, and higher NPQ may lead to excessive excitation energy production, which may enhance the level of ROS production. The energy imbalance may further result in the variation of the redox state of the photosynthetic electron transport chain, triggering the relocation of WHY1 from the chloroplasts to the nucleus, where the signals are transmitted at the gene level.

## 3. Discussion

The pale yellowing phenotype normally appears during plastid dysfunction, such as defective plastid ribosomes, abnormal chlorophyll syntheses, or abnormal plastid RNA processing. As expected, in this study, most of differentially displayed proteins in the *why1/3-var*/WT *oeCIPK14-var*/WT plants were related to plastid dysfunction, such as photosynthesis, amino acid and protein metabolism, or defense and antioxidation.

### 3.1. Proteins Involved in Light-Dependent Reaction of Photosynthesis

The dual-located protein WHY1 has been suggested as an ideal candidate for plastid-to-nucleus retrograde signaling [23]. In chloroplasts, WHY1 is located at the boundary between thylakoids and the nucleoids and, therefore, the WHY1 protein is presumed to be the link between the photosynthetic electron transport chain and gene expression [23]. WHY3 was found to be a cofactor of WHY1, playing a role in plastid genome stability [25] and as an activator of nuclear *kinesin* gene expression [17]. In our study, the results of functional classification and immunoblot analysis revealed that the following seven light reaction-related proteins exhibited greater abundance in the *why1/3-var* and *oeCIPK14-var* plants: Fe–S cluster assembly factor HCF101 (HCF101, spot 1), Magnesium-chelatase subunit ChlI-2 (ChlI-2, spot 4), atpA (spot 3), Chlorophyll a-b binding protein CP26 (CP26, spot 12), PsbP domain-containing protein 4 (PPD4, spot 28), TROL (spot 35), and Cyt f. PPD4 and CP26 are components of PS II [29,30]. CP26 is known as an antenna protein required for the formation of PS II supercomplexes and for the energy transfer from trimeric light-harvesting complex II (LHCII) to the reaction center of PS II [31]. The Cyt b6f complex modulates the electron transfer from PS II to PS I via the quinone pool and plastocyanin [28]. The increase in abundance of Cyt f may accelerate the ETR, which is lower in the *why1/3-var* and *oeCIPK14-var* plants. HCF101 has been shown to serve as a chloroplast scaffold protein for the assembly and transfer of [4Fe–4S] clusters, which are essential for the accumulation of the core complex PS I and soluble ferredoxin-thioredoxin reductases [32]. ATP synthase is responsible for ATP production, and upregulation of atpA was observed in our proteomic data. As expected, we also found that the TROL (spot 35) protein is required for tethering FNR and sustaining efficient linear electron flow (LEF) [33]. The TROL knockout mutant displays a lower ETR and increased NPQ, further confirming our previous results demonstrating that the *why1* line expresses low levels of TROL protein [24]. Consistent with the pale-green phenotype of the two variegated lines, an enzyme for chlorophyll biosynthesis, ChlI-2, showed upregulation. This enzyme catalyzes the first committed step toward chlorophyll synthesis, accompanying Mg^2+^ insertion into protoporphyrin IX and producing Mg-protoporphyrin IX [34]. In addition, another key enzyme for chlorophyll biosynthesis—magnesium protoporphyrin IX methyltransferase (spot 26)—also exhibited upregulation in the *oeCIPK14-var* [35], which suggests that plants may increase the accumulation of chlorophyll and transport it to the two PSs for the synthesis of the LHC complex, thus maintaining the efficiency of electron transfer and alleviating the stress caused by excessive energy. Thylakoid lumenal 29 kDa protein (TL29, spot 54) is located in the thylakoid lumen of chloroplasts [36], and it was identified as a homolog of ascorbate peroxidase, associated with PS II [37]. This protein was found in greater abundance in the *why1/3-var* and *oeCIPK14-var* lines.

### 3.2. Proteins Involved in the Calvin Cycle

The expression of several essential proteins of the Calvin cycle were downregulated in the *why1/3-var* and *oeCIPK14-var* lines, including ribulose bisphosphate carboxylase large chain (RBCL, spots 14, 23, 27, 40), ribulose bisphosphate carboxylase small chain (RBCS, spots 17, 18, 43, 45), ribulose-1,5-bisphosphate carboxylase/oxygenase (Rubisco) activase (RCA, spot 2), and Phosphoglycerate kinase (PGK, spot 5). It is well known that Rubisco—a complex of eight RBCL and eight RBCS subunits containing eight catalytic sites—is the most abundant protein on Earth, utilized by autotrophic organisms to transform CO_2_ into organic compounds via the Calvin–Benson cycle [38]. Rubisco catalyzes the rate-limiting step of photosynthetic carbon reduction for CO_2_ assimilation [38]. In this study, except for spot 32, all other RBCL and RBCS protein levels decreased. In *Arabidopsis*, the activity of Rubisco is inhibited by RCA, which can release ribulose-1,5-bisphosphate (RuBP) from the active sites of Rubisco using the energy from ATP hydrolysis by increasing the ratios of ADP to ATP [39]. This allows CO_2_ to activate the enzyme [39], indicating that the redox state of the photosynthetic electron transport chain has changed, and the production of ATP is inhibited. PGK catalyzes the conversion of 3-phosphoglycerate to 1,3-bisphosphoglycerate, which is a substrate for the synthesis of glyceraldehyde-3-phosphate (G3P) in the Calvin–Benson cycle, where it serves as a substrate for the synthesis of other carbohydrates [40]. Carbonic Anhydrase 1 (CA, spot 59) is located in the chloroplast and catalyzes the conversion of H_2_O and CO_2_ to HCO_3_^−^, which involves CO_2_-dependent stomatal closing [41]. The expression of CA was also decreased in the *why1/3-var* and *oeCIPK14-var* lines, implying a change to the photosynthetic efficiency.

### 3.3. Proteins Associated with Defense and Antioxidation

The results of photosynthetic performance analysis suggest that the *why1/3-var* and *oeCIPK14-var* lines have lower photosynthetic electron transport efficiencies. However, several defense and antioxidation-related proteins (spots 9, 10, 16, 25, 37, 38, 54, and 61) were upregulated in *why1/3-var* and *oeCIPK14-var* for ROS scavenging. Among them, thiamine thiazole synthase (THI, spot 9) and Pyridoxal 5-phosphate synthase subunit PDX1.1 (PDX, spot 37) have shown upregulated expression under oxidative stress [42]. THI (spot 9) is involved in the biosynthesis of the thiamine (vitamin B1) precursor thiazole [43]. PDX (spot 37) catalyzes the formation of pyridoxal 5′-phosphate—a phosphorylated derivative of VB6—which can act as a coenzyme for numerous metabolic enzymes and has been identified as a potent antioxidant [44,45]. The identified protein sAPX (spot 10) and l-ascorbate peroxidase APX1 (spot 25) have been proposed to reduce the generation of ROS and enhance the plant’s tolerance to oxidative stress [46,47]. Additional proteins characterized in this study include: thioredoxin-like protein CDSP32 (spot 38), which has been reported as a thioredoxin and is involved in plastid responses to oxidative stress [48]; the enzyme peptide methionine sulfoxide reductase (PMSR) (spot 61), which catalyzes the reduction of Met sulfoxides back to Met [49], has been shown to repair oxidative damage in chloroplast proteins [48]; VIPP1 (spot 57) is an essential component for thylakoid biogenesis in chloroplasts [50], and its expression is upregulated for membrane maintenance when the membrane integrity of the chloroplast envelope is disturbed [51]. Two members of the glutathione-*S*-transferase (GST) protein family, like glutathione-*S*-transferase F6 (spot 20, GSTF6) and Glutathione-*S*-transferase F7 (spot 60, GSTF7), were identified in this study. GSTF6 (spot 20) was upregulated in *why1/3-var*, but downregulated in *oeCIPK14-var*. GSTF7 was decreased in *why1/3-var*. Taken together, these findings indicate that WHY1/WHY3 or CIPK14 or both are involved in ROS balance. Excessive excitation energy may trigger the accumulation of ROS in chloroplasts in the *why1/3* or *oeCIPK14* lines.

### 3.4. Proteins Related to Protein Metabolism

**Figure 7:**
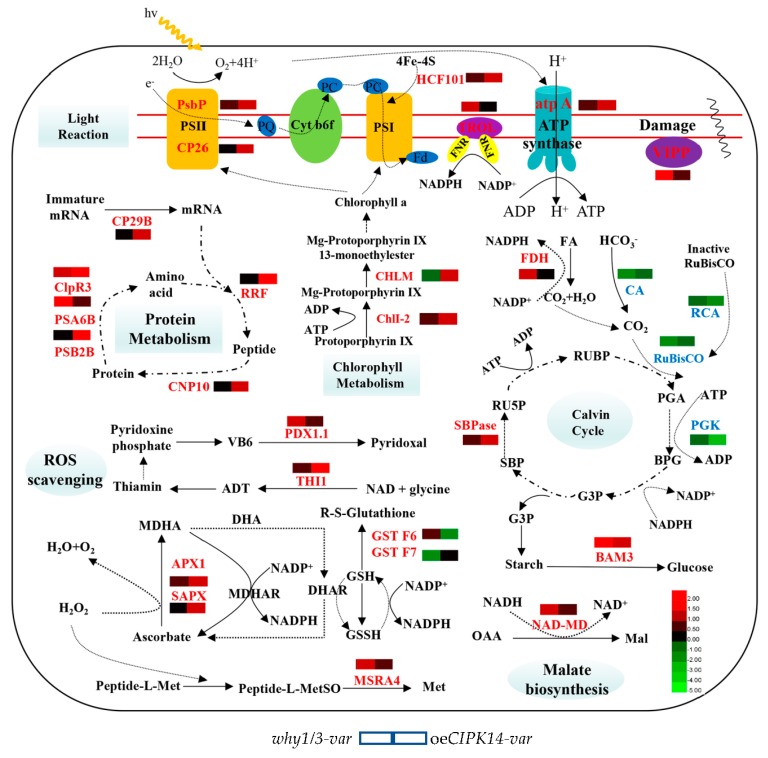
Summary of CIPK14 or/and WHIRLY1/3 mediated pathways in chloroplasts, including light reaction, chlorophyll metabolism, ROS scavenging, malate biosynthesis, calvin cycle, and protein metabolism. The solid lines indicate a direct pathway, the dashed lines indicate a hypothetic connection.

Intriguingly, in the present study, several identified protein spots were found to be involved in protein metabolism. Among them, five spots (spots 11, 19, 29, 30, and 39) showed an increased abundance in the *why1/3-var* and *oeCIPK14-var* lines. For example, the ATP-dependent Clp protease is a plastid protease that plays an essential role in chloroplast development and maintenance [52,53]. ATP-dependent Clp protease proteolytic subunit-related protein 3 (spot 11) was upregulated in the *why1/3-var* and *oeCIPK14-var* lines, indicating chloroplast dysfunction. In general, selective proteolysis in plants is largely mediated by the ubiquitin (Ub)/26S proteasome system [54], where abnormal polypeptides are marked by the covalent attachment of Ub and are degraded by the 26S proteasome [55], which is composed of two subparticles—the 20S core protease and the 18S regulatory particle [55]. The 20S core protease subunit beta type-2-B (spot 29) and proteasome subunit alpha type-6-B (spot 39) [55] and protease subunit alpha type-6-A (spot 13) were all downregulated in the *why1/3-var* and *oeCIPK14-var* lines. Ribosome-recycling factor (spot 30, RRF), located in the chloroplast [56], increased in the *oeCIPK14-var* lines; RRF is essential for embryogenesis and chloroplast biogenesis, and is thought to be involved in the translation of chloroplast proteins [56]. The chaperonin CNP10 (spot 19) was also shown to localize to the chloroplasts [57]. It was upregulated in *oeCIPK14-var*, and is essential for proteins involved in cellular protein folding [57]. In addition, our data show that RNA-binding protein CP29B (spot 8) was upregulated in the *oeCIPK14-var*; CP29B is a kind of a chloroplast RNA-binding protein and may be involved in the processing of chloroplast RNA [58]. These results demonstrate that CIPK14-mediated plastid WHY1/WHY3 proteins might be involved in plastid protein metabolism, including protein translation, synthesis, and proteolysis (Figure 7).

Although the trends in altered transcription levels of the encoding genes of these proteins were not fully confirmed, on the one hand, the discrepancy between mRNA and protein levels may be caused by the different half-lives of protein and mRNA, or post-transcriptional or post-translational processing and modification. In fact, several identified protein spots are involved in protein metabolism or post-transcriptional or post-translational processing and modification. On the other hand, it seems that the alteration of the transcriptional levels in the *why1/3-var* and *oeCIPK14-var* lines could not be reverted in the *oeWHY1* or *cipk14* mutants. It has been proposed that, in maize, *why1* plants with the pale yellowing phenotype may be related to impaired RNA processing [19,20]; however, in *Arabidopsis*, only double-mutated *why1/3* showed the pale yellowing phenotype [25]. This suggests that the WHY1 protein in monocotyledon plants shares functions with both WHY1 and WHY3 in dicotyledonous *Arabidopsis*. This will be addressed by genetic rescue experiments in the future. It is not unexpected that the *cipk14* plant cannot recused the leaf pale yellowing phenotype of *oeCIPK14-var* and the gene expression pattern of *oeCIPK14-var*. Actually, CIPK14 mainly acts as a kinase, phosphorylating WHY1 and thus inducing WHY1 to enter the nucleus; *cipk14* mostly shows the staygreen phenotype [28], with only 5% of the plants showing pale yellowing. The pale yellowing of *oeCIPK14-var* leaves resulting from WHY1/WHY3 deficiency in plastids can be rescued by overexpressing the plastid isoform of WHY1 [28]. In fact, in this study, five proteins (ClpR3, RBL, RBS, RRF, and BAM3) showed altered abundance in the *why1/3-var* and *oeCIPK14-var* lines; interestingly, their mutants have been reported showing similar leaf yellowing and small rosette phenotypes [53,56,59]. The speculated mechanism of action connecting protein metabolism with the CIPK14 and WHIRLY protein-mediated pale yellowing of leaves will be addressed in future studies. 

## 4. Materials and Methods

### 4.1. Plant Materials and Growth Conditions

Plants of *Arabidopsis thaliana* Columbia ecotype and the mutants were cultivated in a vermiculite matrix after vernalization. Plants were grown in a controlled climatic chamber at 13/11 h light/dark cycle with a periodic temperature of 22 °C/18 °C, a light intensity of 60 μmol·m^−2^s^−1^, and a relative humidity of 65%. The rosette leaves of each plant genotype were collected at 4 weeks after germination. The samples were frozen in liquid nitrogen and stored at −80 °C before use. Three biological replicates were used for each experiment.

T-DNA insertion lines SALK_023713 (*why1*) for WHY1 and SALK_009699 (*cipk14*) for CIPK14 were provided by NASC. Seeds of the *WHY1* and *WHY3* double-mutant (*why1/3*) were kindly provided by Normand Brisson, Department of Biochemistry, Montreal University, Montreal, Canada. Overexpression *WHY1* (*oeWHY1*) and *CIPK14* (*oeCIPK14*) lines were prepared from previous work [28].

### 4.2. Chlorophyll Content Measurement and Photosynthetic Parameters Analysis

Colored threads were used for labeling rosette leaves after their emergence, as previously described [8]. The weight of five leaves from 12 independent 4-week-old plants were measured, and each leaf was mixed with 1 mL 95% ethanol (*v*/*v*) in a 1.5 milliliter (mL) Eppendorf tube. Pigments were extracted after incubating for 48 h in the dark. The absorbance of the extracts was measured at 470, 649, and 665 nm by the Flexstation 3 Microplate Reader (Molecular Devices, Silicon Valley, CA, USA), and the total chlorophyll content was determined according the method described in [60].

Chlorophyll fluorescence was measured, and the chlorophyll fluorescence image was taken using an Imaging-PAM-Maxi (Walz, Effeltrich, Germany) as described by Shao [61]. Five leaves from 4-week-old plants were selected for measurement after 30 min adaptation to darkness. The minimal fluorescence yield (F_o_) was measured at a low light intensity, and the maximal fluorescence yield (F_m_) was measured under saturation pulse. Then, all of the leaves were exposed to a light intensity of 54 μmol·m^−2^s^−1^ photosynthetically active radiation (PAR), the kinetic curves were gained according to the manufacturer’s instructions for the instrument. The maximum photochemical efficiency of photosystem II (F_v_/F_m_), the nonphotochemical quenching (NPQ) of photosystem II fluorescence, and the electron transport rate (ETR) were calculated by control software. The chlorophyll fluorescence images of whole plants were taken after 30 min adaptation to darkness. Through one saturation pulse, the values of F_o_ and F_v_/F_m_ were calculated, and fluorescence images were viewed using Imaging-PAM-Maxi (Walz, Effeltrich, Germany).

### 4.3. Protein Extraction and 2-DE Analysis

The total protein of rosette leaves was extracted by the phenol method [62]. Briefly, approximately 3 g material was ground with liquid nitrogen, then suspended in 12 mL ice-cold extraction buffer (50 mM PBS (PH 7.8), 5 mM EDTA, 2% (*v*/*v*) β-mercaptoethanol, 0.5% (*v*/*v*) NP-40, 1 mM Phenylmethane-sulfonyl fluoride (PMSF), 1% (*w*/*v*) polyvinylpolypyrrolidone (PVPP)) on ice and vortexed for 5 min. An equal volume of ice-cold Tris-saturation phenol (pH 8.0) was added to the suspension and vortexed for 10 min. The phenol phase was collected after centrifugation (4 °C, 15 min, 16,000× *g*), and proteins were precipitated overnight with five volumes of 0.1 M methanol/ammonium acetate at −20 °C. The protein pellets were collected after centrifugation (4 °C, 15 min, 16,000× *g*) and rinsed three times in ice-cold acetone/13 mM dithiothreitol (DTT). Between each wash, the proteins were incubated for 1 h at −20 °C. After centrifugation (4 °C, 15 min, 16,000× *g*), the supernatant was discarded carefully, and the protein pellets were air-dried.

The protein pellets were dissolved in sample lysis buffer (7 M urea, 2 M thiourea, 4% CHAPS, 65 mM DTT, 2% IPG Buffer pH 3–10 NL), and protein concentrations were determined by Bradford method [63]. A total of 1.5 mg protein was loaded onto an IPG Strip (24 cm, 3–10 NL) and rehydrated over 12 h at 25 °C. An Ettan IPGphor system (GE Healthcare, Uppsala, Sweden) was employed for Isoelectric focusing (IEF) using the program: 30 V (0.5 h), 100 V (1 h), 200 V (1 h), 500 V (3 h), 1 kV (1 h), 10 kV (2 h, gradient), and, finally, 10 kV up to 60,000 Vhs. Then, the strips were equilibrated for 15 min in equilibration buffer I (50 mM Tris–HCl (pH 8.8), 6 M urea, 30% (*v*/*v*) glycerol, 2% (*w*/*v*) SDS, 1% DTT) and in equilibration buffer II (50 mM Tris–HCl (pH 8.8), 6 M urea, 30% (*v*/*v*) glycerol, 2% (*w*/*v*) SDS, 2.5% iodoacetamide) for 15 min with gentle shaking. After equilibration, the strips were transferred to 12.5% SDS-PAGE, and electrophoresis was performed with an Ettan DALT-six System (GE Healthcare, Chicago, IL, USA) at 10 mA per gel for 1 h and then 15 mA per gel overnight. After electrophoresis, the 2-DE gels were stained by Coomassie Brilliant Blue R250 (CBB R250). Three independent biological replicates were used for each genotype.

### 4.4. Data Analysis In-Gel Protein Digestion and Protein Identification

Gels were scanned at a resolution of 600 dpi with a scanner (EPSON Expression 11000XT, Suwa, Japan), and the images were quantitatively analyzed by ImageMaster^TM^ 2D Platinum 7.0 software (GE Healthcare, Uppsala, Sweden). The differential protein spots were in-gel digested and identified according to the methods as described [64]. In short, the protein spots were excised from the CCB R250-stained gels and subjected to in-gel trypsin digestion for 12 h at 37 °C. Peptides were extracted twice in 50% ACN/0.1% TFA, then the extracts were dried completely by a vacuum centrifuge. Peptide mixtures were dissolved in 0.1% TFA, and 0.8 μL of the peptide solution was mixed with 0.4 μL matrix α-cyano-4-hydroxycinnamic acid in 30% ACN/0.1% TFA before spotting on the target plate. An AB SCIEX MALDI TOF-TOFTM 5800 Analyzer (AB SCIEX, Foster City, CA, USA) equipped with a neodymium:yttrium-aluminum-garnet laser (laser wavelength was 349 nm) was employed for acquiring peptide mass fingerprints (PMFs). All automatic data analyses and database searches were conducted using GPS Explorer TM software (version 3.6, AB SCIEX) running a mascot search algorithm (v2.3, Matrix Science, London, UK) for protein identification. Proteins with a protein score confidence interval (CI) above 95% (protein score > 61) were considered confident identifications. The identified proteins were then matched to specific processes or functions by searching Uniprot.

### 4.5. RNA Extraction, Reverse Transcription, and qRT-PCR Analysis

Total RNA was extracted from the 4-week-old rosette leaves of *Arabidopsis* using Trizol Reagents (TransZol up, TRANSGEN BIOTECH, Beijing, China) according to the manufacturer’s protocol. First-strand cDNA was synthesized using cDNA Synthesis SuperMix with gDNA Removal (TRANSGEN BIOTECH, Beijing, China) according to the manufacturer’s instructions. Specific oligonucleotide primers were designed by http://quantprime.mpimp-golm.mpg.de and synthesized by Sangon Biotech (Shanghai, China). Quantitative real-time PCR was performed using the Ultra SYBR Mixture (CWBIO, Beijing, China) according to the manufacturer’s instructions using a Lightcycler^®^ 96 (Roche, Basel, Switzerland). *GAPC2* was used as an internal standard, and relative gene expression was analyzed using the 2^−ΔΔ*C*T^ Method. The primer pairs are shown in Supplemental Table S1.

### 4.6. Immunological Analyses

Proteins were extracted from rosette leaves according to the phenol method [62]. Equivalent quantities of proteins were determined by the Bradford method [63]. Proteins were separated on 12.5% polyacrylamide gels [65], transferred to PVDF membranes by semi-dry electroblotting, and immunodetected according to protocols as described in [24]. Primary antibodies directed toward RBCL, RBCS, and PSBR were purchased from Agrisera (Vännäs, Sweden).

## 5. Conclusions

We conclude that in the *why1/3-var* or *oeCIPK14-var* lines, most differentially expressed proteins are involved in photosynthesis, amino acid and protein metabolism, or defense and antioxidation (Figure 7). They are related to photosynthesis and the response to insufficient energy supply. The regulation of CIPK14 phosphorylation-mediated WHIRLY1/WHIRLY3 deficiency in plastids is speculated to be controlled by a mechanism of amino acid and plastid protein metabolism at the post-transcriptional level. The details of this mechanism will be addressed in future studies.

## Figures and Tables

**Table 1:** Differential proteins identified by MALDI-TOF/TOF-MS of *why1/3-var* and *CIPK14-var*.

Spot No ^a^	Protein Name	Accession No ^b^	Mascot Score	Matched Peptides	Theor MW (kDa)/pI ^c^	Cov% ^d^	Subcellular Loc ^e^
**Light reaction**							
1	Fe-S cluster assembly factor HCF101	HF101_ARATH	371	13	57.728/5.91	20%	plastid
2	RCA	F4IVZ7_ARATH	499	18	48.469/7.55	34%	chloroplast
3	ATP synthase subunit alpha	ATPA_ARATH	927	32	55.294/5.19	50%	plastid
4	Magnesium-chelatase subunit ChlI-2	CHLI2_ARATH	365	23	46.069/5.36	44%	chloroplast
12	Chlorophyll a-b binding protein CP26	CB5_ARATH	213	12	30.183/6	35%	plastid
26	Magnesium protoporphyrin IX methyltransferase	CHLM_ARATH	653	23	33.775/7.68	53%	chloroplast
28	PsbP domain-containing protein 4	PPD4_ARATH	122	8	28.484/7.02	33%	plastid
33	Oxygen-evolving enhancer protein 3-2	PSBQ2_ARATH	464	16	24.628/9.72	59%	plastid
34	PSBR	A0A178WGP6_ARATH	112	7	9.77/10.1	39%	plastid
35	TROL	A0A178V0X3_ARATH	444	21	54.448/5.09	29%	chloroplast
**Calvin cycle**							
5	Phosphoglycerate kinase 1	PGKH1_ARATH	642	24	50.081/5.91	37%	chloroplast
6	Sedoheptulose-1,7-bisphosphatase	S17P_ARATH	354	18	42.388/6.17	28%	chloroplast
14	Ribulose bisphosphate carboxylase large chain (Fragment)	A0A142I795_ARATH	292	12	51.833/6.17	9%	chloroplast
17	Ribulose bisphosphate carboxylase small chain	A0A178UL15_ARATH	326	14	20.33/7.59	45%	chloroplast
18	Ribulose bisphosphate carboxylase small chain 1A	RBS1A_ARATH	369	15	20.203/7.59	45%	chloroplast
23	Ribulose bisphosphate carboxylase large chain (Fragment)	A0A142I795_ARATH	292	12	51.833/6.17	18%	plastid
27	Ribulose bisphosphate carboxylase large chain	RBL_ARATH	740	31	52.922/5.88	42%	chloroplast
32	Ribulose bisphosphate carboxylase small chain	A0A178UL15_ARATH	326	14	20.33/7.59	45%	chloroplast
40	Ribulose bisphosphate carboxylase large chain (Fragment)	A0A142I795_ARATH	292	12	51.833/6.17	26%	chloroplast
43	Ribulose bisphosphate carboxylase small chain 1A	RBS1A_ARATH	362	13	20.203/7.79	49%	chloroplast
45	Ribulose bisphosphate carboxylase small chain 1B	RBS1B_ARATH	348	14	18.506/8.22	53%	chloroplast
59	Beta carbonic anhydrase 1	BCA1_ARATH	307	15	37.426/5.74	48%	plastid/ cytomembrane
**Defense and antioxidation**							
7	Glucan endo-1,3-beta-glucosidase, acidic isoform	E13A_ARATH	568	16	37.316/4.85	33%	secretion
9	Thiamine thiazole synthase	THI4_ARATH	183	10	36.641/5.82	28%	plastid
10	SAPX	A0A178V0Q5_ARATH	524	24	40.446/8.31	47%	chloroplast
16	Glycine-rich RNA-binding protein 7	RBG7_ARATH	167	9	16.88/5.85	51%	cytoplasm/nucleus
20	Glutathione S-transferase F6	GSTF6_ARATH	355	15	23.471/5.8	49%	cytoplasm
24	V-type proton ATPase subunit E1	VATE1_ARATH	328	30	26.044/6.04	75%	vacuole
25	l-ascorbate peroxidase 1	F4HU93_ARATH	389	16	27.503/5.85	52%	cytoplasm
37	Pyridoxal 5′-phosphate synthase subunit PDX1.1	PDX11_ARATH	423	23	32.841/5.75	37%	cytoplasm
38	Thioredoxin-like protein CDSP32	CDSP_ARATH	371	20	33.663/8.65	35%	chloroplast
44	GRP7	A0A178VQY8_ARATH	315	9	16.937/5.85	37%	cytoplasm/nucleus
46	V-type proton ATPase subunit G1	VATG1_ARATH	304	10	12.389/5.77	70%	vacuole
51	Formate dehydrogenase	FDH_ARATH	354	18	42.383/7.12	36%	chloroplast/mitochondria
52	12-oxophytodienoate reductase 3	OPR3_ARATH	782	24	42.664/7.71	54%	peroxysome
54	Thylakoid lumenal 29 kDa protein	TL29_ARATH	475	24	37.911/8.59	53%	plastid
55	Remorin	REMO_ARATH	427	26	20.955/8.63	65%	plasmalemma
57	VIPP1	A0A178W0D3_ARATH	412	23	28.895/5.9	67%	plastid
60	Glutathione S-transferase F7	GSTF7_ARATH	552	18	23.583/6.14	52%	cytoplasm
61	Peptide methionine sulfoxide reductase A4	MSRA4_ARATH	236	12	38.626/8.96	26%	plastid
**Amino acid metabolism**							
22	Serine hydroxymethyltransferase 4	GLYC4_ARATH	504	22	51.685/6.8	46%	cytoplasm
48	Asparagine synthetase [glutamine-hydrolyzing] 2	ASNS2_ARATH	235	18	64.989/6.01	27%	cytoplasm/plasmodesmata
50	Glutamate-glyoxylate aminotransferase 1	GGT1_ARATH	478	20	53.267/6.49	37%	peroxysome
58	Acetylglutamate kinase	NAGK_ARATH	424	14	36.572/9.04	36%	plastid
**Proteometabolism**							
11	ATP-dependent Clp protease proteolytic subunit-related protein 3	CLPR3_ARATH	597	23	36.284/8.64	41%	chloroplast
13	Proteasome subunit alpha type-6-A	PSA6A_ARATH	926	25	27.277/5.6	58%	cytoplasm/nucleus
15	30S ribosomal protein S6 alpha	RR6_ARATH	119	8	22.746/5.92	26%	plastid
19	CPN10	O80504_ARATH	567	10	15.04/8.75	45%	chloroplast
29	Proteasome subunit beta type-2-B	PSB2B_ARATH	715	22	21.97/6.21	70%	cytoplasm/nucleus
30	Ribosome-recycling factor	RRFC_ARATH	497	18	30.403/9.46	44%	plastid
31	Peptidyl-prolyl cis-trans isomerase CYP18-3	CP18C_ARATH	307	11	18.361/7.68	34%	cytoplasm
39	Proteasome subunit alpha type-6-B	PSA6B_ARATH	312	19	27.333/5.75	56%	cytoplasm/nucleus
56	Peptidyl-prolyl cis-trans isomerase CYP19-1	CP19A_ARATH	406	14	18.48/8.65	43%	cytoplasm
62	Nascent polypeptide-associated complex subunit beta	A0A178W6R8_ARATH	332	14	16.935/5.50	45%	cytoplasm/nucleus
**Energy metabolism**							
21	Beta-amylase 3	BAM3_ARATH	510	24	61.314/6.59	41%	chloroplast
**Malate biosynthesis**							
36	Malate dehydrogenase	MDHP_ARATH	334	12	42.379/8.66	23%	chloroplast
47	NADP-dependent malic enzyme 2	MAOP2_ARATH	323	19	64.372/6.01	25%	cytoplasm
49	l-galactono-1,4-lactone dehydrogenase	GLDH_ARATH	759	35	68.513/8.7	38%	mitochondria
**Lipid metabolism**							
53	GDSL esterase/lipase At1g29670	GDL15_ARATH	841	19	39.847/8.85	44%	secretion
**Transcription**							
8	RNA-binding protein CP29B	CP29B_ARATH	602	16	30.699/5.06	52%	plastid
**Unknow function**							
41	At1g13930/F16A14.27	Q9XI93_ARATH	310	14	16.154/4.82	87%	chloroplast/plasmalemma
42	Kinesin-like calmodulin-binding protein	KCBP_ARATH	54	35	143.359/6.69	21%	cytoplasm

^a^ Numbering corresponds to 2-DE gel in Figure 1A–C. ^b^ Database accession of the identified proteins in uniprot (http://www.uniprot.org/). ^c^ Molecular mass and pI theoretical. ^d^ Percentage of predicted protein sequence with match sequence. ^e^ Subcellular localization of the identified protein base on uniprot and previous literature.

## Supplementary Materials

Supplementary materials can be found at http://www.mdpi.com/1422-0067/19/8/2231/s1.

## Abbreviations

ADP	adenosine diphosphate
ADT	adenosine diphosphate 5-(2-hydroxyethyl)-4-methylthiazole-2-carboxylic acid
APX1	l-ascorbate peroxidase 1
ATP	adenosine triphosphate
ATPA	ATP synthase alpha subunit
BAM3	beta-amylase 3
BPG	1,3-diphosphoglycerate
CA	carbonic anhydrase
ChlI-2	magnesium-chelatase subunit ChlI-2
CHLM	magnesium protoporphyrin IX methyltransferase
CLPR3	ATP-dependent Clp protease proteolytic subunit-related protein 3
CNP10	10 kDa chaperonin
CP26	chlorophyll a-b binding protein CP26
CP29B	RNA-binding protein CP29B
Cyt b6f	cytochrome b6f
DHAR	dehydroascorbate reductase
FA	formate
Fd	ferredoxin
FDH	formate dehydrogenase
FNR	ferredoxin-NADP (H) oxidoreductase
G3P	glyceraldehydes-3-phosphate
GSH	reduced glutathione
GSSH	oxidized glutathione
GST	glutathione S-transferase
HCF101	Fe-S cluster assembly factor HCF101
Mal	malate
MD	malate dehydrogenase
MDHA	monodehydroascorbate
MDHAR	MDHA reductase
Met	methionine
MetSO	methionine sulfoxide
MSRA4	peptide methionine sulfoxide reductase A4
NADP^+^/NADPH	nicotinamide adenine dinucleotide phosphate
NAD^+^/NADH	nicotinamide adenine dinucleotide
OAA	oxaloacetic acid
PC	plastocyanin
PDX1.1	pyridoxal 5′-phosphate synthase subunit PDX1.1
PGA	3-phosphoglycerate
PGK	phosphoglycerate kinase
PQ	plastoquinone
PSI	photosystem I
PSII	photosystem II
PSA6B	proteasome subunit alpha type-6-B
PSB2B	proteasome subunit beta type-2-B
PsbP	PsbP domain-containing protein 4
RCA	ribulose-1,5-bisphosphate carboxylase/oxygenase activase
RRF	ribosome-recycling factor
Ru5P	ribulose-5-phosphate
RuBisCO	ribulose-1,5-bisphosphate carboxylase/oxygenase
THI	thiamine thiazole synthase
TROL	thylakoid rhodanese-like protein
SBP	sedoheptulose-1,7-bisphosphate
SBPase	sedoheptulose-1,7-bisphosphatase
SAPX	stromal ascorbate peroxidase
VB6	vitamin B6
VIPP1	vesicle-inducing protein in plastids
