# Supplementary material for: Comparative Proteomic Analysis of Coregulation of CIPK14 and WHIRLY1/3 Mediated Pale Yellowing of Leaves in Arabidopsis
Source: Int J Mol Sci. 2018 Jul 31;19(8):2231. doi: 10.3390/ijms19082231 (PMC6121582; doi:10.3390/ijms19082231)
Supplement: Supplementary file 1 [file ijms-19-02231-s001.pdf]

Supplemental Table S1 The list of primer sequences for qRT-PCR

| TAIR number | Gene name | Primer sequences（5' to 3'） |
|---|---|---|
| At1g14410 | WHIRLY1 | FP: TTTTACGTGGGTCATTCGAT<br>RP: GTCCACTGTTAACGCAGCTT |
| At2g02740 | WHIRLY3 | FP: ACGGTGAAGTCTCGACAAAG<br>RP: ATCGAATGACCCACGTAAAA |
| AT5G01820 | CIPK14 | FP:TAGACACGAATCCGCAGACGA<br>RP:TCGTCGTAGCCCTGTTTGAACC |
| AT1g13440 | GAPC2 | FP:ACCACTGTCCACTCTATCACTGA<br>RP:TGAGGGATGGCAACACTTTCCC |
| AT3G24430 | spot 1 | FP:CACTTTGACGCTGATGGGAAACG<br>RP:TGCCGAATTGCTTGACCACCTC |
| AT2G39730 | spot 2 | FP:AGACCGTATCGGTGTCTGCAAG<br>RP:CCCTCAAAGCACCGAAGAAATCG |
| ATCG00120 | spot 3 | FP:TTCTTCCGTGGCTCAGGTAGTG<br>RP:GGCCGTTTCAGCTACCACAATAG |
| AT5G45930 | spot 4 | FP:TGTGCTGAGCTGGACGTTGATG<br>RP:AGCGCTCTAGCTGCTCTGTTTATC |
| AT3G12780 | spot 5 | FP:CGAGCTTCTTGGTATTGAGGTCAC<br>RP:GGTAGAGAAGCCACCAAGCTTTCC |
| AT3G55800 | spot 6 | FP:TCGACAACTCCGAATACAGCAAGC<br>RP:AACCATTCCTCCGGTGTATCGC |
| AT3G57260 | spot 7 | FP:AGCTTCCTTCTTCAACCACACAGC<br>RP:TGGCAAGGTATCGCCTAGCATC |
| AT2G37220 | spot 8 | FP:GCTCAGCAGTTCAATGGCTATGAG<br>RP:ACCATCTTCCCTCTTTGGTGGTG |
| AT5G54770 | spot 9 | FP:TTTCTCCGCCATGATTGTTCGC<br>RP:AAGCCACACCAATCTCGTCAAGG |
| AT4G08390 | spot 10 | FP:TCTAGGCCAGAACGTAGTGGTTGG<br>RP:TGCTCCAGGTCCTTCTTTCGTG |
| AT1G09130 | spot 11 | FP:TATGATGCCACATGCCAAAGCG<br>RP:ACATCACTGGCAGGCATCAACC |
| AT4G10340 | spot 12 | FP:GCCGTAGTTGCTGAGGTTGTTC<br>RP:AGCTTGTCCTCGAAATCCAATCCG |
| At5g35590 | spot 13 | FP:AGGTCGTCTCTTCCAAGTCGAG<br>RP:TTCTGCGTAACGACGCATACTG |
| AT1G64510 | spot 15 | FP:CATGTCTGAAGATGAACGGCTTGG<br>RP:TCCTGCCACAAGCAACTCTTCG |
| AT2G44650 | spot 19 | FP:GGACAACAAGTTGGACCTGGAAAG<br>RP:CATCGGTTCCCAAATCGACCTC |

| TAIR number | Gene name | Primer sequences（5’ to 3’） |
|---|---|---|
| AT1G02930 | spot 20 | FP:AAGAGCCTTTCATCCTTCGCAACC<br>RP:TGTCCTTGCCAGTTGAGAGAAGG |
| AT4G17090 | spot 21 | FP:AAAGCACGGTCTCAAACTCCAG<br>RP:ACTGCAAGAGTCTCCTACGTTTCC |
| AT4G13930 | spot 22 | FP:AAAGCCAATGCTGTTGCCCTTG<br>RP:CAGAGCTTCTCAACCTTGTTTCCG |
| AT4G11150 | spot 24 | FP:TGCGCGTCTTGATGTGGCATTC<br>RP:GCCGAACAACGACTTACGGATCAC |
| AT1G07890 | spot 25 | FP:TTTCCACCCTGGAAGAGAGGAC<br>RP:TGGTCACAACCCTTGGTAGCATC |
| AT4G25080 | spot 26 | FP:AGGAGCAATCGTCTCTGCTTCC<br>RP:ATGGTAGTTGTGCCTTTGCCTTC |
| AT1G77090 | spot 28 | FP:CGAACCTGATGAAGAAGGTTGGAG<br>RP:ACCAAGATCAGCGATCGATACAGG |
| AT4G14800 | spot 29 | FP:GGCGGATACATCAGCAGTTCACAG<br>RP:TCCGTAAACTGAACCCGGTCAC |
| AT3G63190 | spot 30 | FP:GCGATGCTTGACAAGATTGAGGTG<br>RP:CCGGAGTACTGATTTGGGCTATGC |
| AT4G38740 | spot 31 | FP:AGACGTGGTAAAGGCCATCGAG<br>RP:ATCGGCAACAACCACAGGCTTC |
| AT4G05180 | spot 33 | FP:ACACCGTTATCTCCGCTAAGCC<br>RP:TGATCTCGCCGCATAGTCCAAG |
| AT1G79040 | spot 34 | FP:TAACGGCAGCATGGACTTGAGG<br>RP:ACTTGTAAACACCGTATCCCTTGC |
| AT4G01050 | spot 35 | FP:ACCAACCGAAACAGAAGCCAAAC<br>RP:ATGGAGGCTTCAAGTCAGGGTACG |
| AT3G47520 | spot 36 | FP:GCTCACTGTTAGGATTCAGAACGC<br>RP:AACCTGCACCTGCCTTAGCATC |
| AT2G38230 | spot 37 | FP:AAGGCGGTGTTGCTCGAATGAG<br>RP:TTTCGCCATCACCGGAATCGTC |
| AT1G76080 | spot 38 | FP:TTCTTGCTCGTCATGGGTGATG<br>RP:CTGGTCCGGTTCAATACCTTCTTC |
| AT2G05840 | spot 39 | FP:AGGTCGTCTCTTCCAAGTCGAG<br>RP:TTCTGCGTAACGACGCATACTG |
| AT2G21660 | spot 44 | FP:TCAATGGCGTCCGGTGATGTTG<br>RP:TCATCAGTGGCCCATGCTAGAC |
| AT3G01390 | spot 46 | FP:AGAGGAAACTTGAGGAGACAAGC<br>RP:CTTGCTCCAGCCTCTTCACATTCG |

| TAIR number | Gene name | Primer sequences（5’ to 3’） |
|---|---|---|
| AT5G11670 | spot 47 | FP:GGACCTCATTGGTGCTGTTAATGC<br>RP:CCACACCGGATGTTCCAATGAG |
| AT5G65010 | spot 48 | FP:TGTCTTCCCGGATAACACACCTC<br>RP:AGTCGCTCTAGCAGCACTCTTC |
| AT3G47930 | spot 49 | FP:GGCCACCTAAGGACAAACCAAAG<br>RP:CCTGGACCCTATACTTCACAATGC |
| AT1G23310 | spot 50 | FP:TTACCAACGGGAGCTCTCCAAG<br>RP:GTTGTCCTCAGATGGAACACACC |
| AT5G14780 | spot 51 | FP:AAGGAAGGCCCTGATTGCGAAC<br>RP:AAGGGAGTGGAGATTAGGACGTG |
| AT2G06050 | spot 52 | FP:ACGTGCTTCTCATGCAGTGTATC<br>RP:TACTTCACGTGGGAACCATCGG |
| AT1G29670 | spot 53 | FP:AGAAGAAACCGGTCGACAATTGGG<br>RP:GCTGCACAACTTGCGATACTGTG |
| AT4G09010 | spot 54 | FP:GCATATGGTTCAGCTGGTCAGTGG<br>RP:TCAGCCTCTGTTGCATCACTCC |
| AT2G45820 | spot 55 | FP:TCTGGTTCGGCCGATAGAGATG<br>RP:TTCCCAAGCATGCACATCAGAG |
| AT2G16600 | spot 56 | FP:ACATGACCGTCGGTGGCAAATC<br>RP:TTCTCGGCGGTTTCTGGTGTTG |
| AT1G65260 | spot 57 | FP:TCTTGCACGTGAGGCCCTTAAAC<br>RP:TTCAAAGCAGTAGCGTTGTCAGC |
| AT3G57560 | spot 58 | FP:ATACAGTTGCTGGAGAGCTTGCG<br>RP:TTCCAGCCACATCAGTCAGCAG |
| AT3G01500 | spot 59 | FP:AGATGCCTTCGTGGTCCGTAAC<br>RP:CAACGCCACCGTATTTGACCTTG |
| AT1G02920 | spot 60 | FP:AGCCTTTCATCTTCCGCAACCC<br>RP:TTGGAGCCAAGGGAGACAAGTTGG |
| AT4G25130 | spot 61 | FP:ACTGGCACTACGGGACATAACG<br>RP:ACCCTGACGATTCAAGGTGGTTG |
| AT1G73230 | spot 62 | FP:ATCAGCCAACTTGGACCAGATAAC<br>RP:ACCTGGAGCTTGTTTCTGGAATTG |
